# Does the level of inferior mesenteric artery ligation affect short-term and long-term outcomes of patients with sigmoid colon cancer or rectal cancer? A single-center retrospective study

**DOI:** 10.1186/s12957-022-02741-9

**Published:** 2022-09-01

**Authors:** Yawei Wang, Yan Wang, Liaonan Zou, Lingna Deng, Tianchong Wu, Linsen Liu, Jiling Jiang, Tailai An

**Affiliations:** 1 The First Department of Surgery, Shenzhen Traditional Chinese Medicine Hospital, Fuhua Road 1, Futian District, Shenzhen, 518033 Guangdong People’s Republic of China; 2 Department of Radiology, Shenzhen People’s Hospital, Dongmen Road 1017, Luohu District, Shenzhen, 518020 Guangdong People’s Republic of China; 3 Department of General Surgery, The Affiliated Zhuhai Hospital, Guangdong Provincial Hospital of Traditional Chinese Medicine, Jingle Road 52, Xiangzhou District, Zhuhai, 519015 Guangdong China; 4 Department of Gastrointestinal Surgery, Guangdong Provincial Hospital of Traditional Chinese Medicine, Dade Road 111, Yuexiu District, Guangzhou, 510006 Guangdong China; 5 Department of Pathology, Qingyuan People’s Hospital, Yinquan Road B24, Qingcheng District, 511518 Qingyuan, Guangdong People’s Republic of China; 6 Department of Hepatobiliary and Pancreatic Surgery, Shenzhen People’s Hospital, Dongmen Road 1017, Luohu District, Shenzhen, 518020 Guangdong People’s Republic of China

**Keywords:** Inferior mesenteric artery, High tie, Low tie, Short-term and long-term outcomes, Laparoscopic surgery, Anastomotic fistula

## Abstract

**Background:**

For sigmoid colon or rectal cancer, a definite consensus regarding the optimal level ligating the inferior mesenteric artery (IMA) has not been reached. We performed this study to determine whether the ligation level significantly affected short-term and long-term outcomes of patients with sigmoid colon or rectal cancer after curative laparoscopic surgery.

**Methods:**

Medical records of patients with sigmoid colon or rectal cancer who had undergone curative laparoscopic surgery between January 2008 and December 2014 at the Department of Gastrointestinal Surgery, Guangdong Provincial Hospital of Traditional Chinese Medicine were reviewed. Then, the high tie group (HTG) was compared with the low tie group (LTG) in terms of short-term and long-term outcomes.

**Results:**

Five-hundred ninety patients were included. No significant differences between two groups regarding baseline characteristics existed. HTG had a significantly higher risk of anastomotic fistula than LTG (21/283 vs 11/307, *P* = 0.040). Additionally, high ligation was proven by multivariate logistic regression analysis to be an independent factor for anastomotic fistula (*P* = 0.038, *OR* = 2.232, 95% *CI*: 1.047–4.758). Furthermore, LT resulted in better preserved urinary function. However, LTG was not significantly different from HTG regarding operative time (*P* = 0.075), blood transfusion (*P* = 1.000), estimated blood loss (*P* = 0.239), 30-day mortality (*P* = 1.000), ICU stay (*P* = 0.674), postoperative hospital stay (days) (*P* = 0.636), bowel obstruction (*P* = 0.659), ileus (*P* = 0.637), surgical site infection (SSI) (*P* = 0.121), number of retrieved lymph nodes (*P* = 0.501), and number of metastatic lymph nodes (*P* = 0.131). Subsequently, it was revealed that level of IMA ligation did not significantly influence overall survival (OS) (*P* = 0.474) and relapse-free survival (RFS) (*P* = 0.722). Additionally, it was revealed that ligation level did not significantly affect OS (*P* = 0.460) and RFS (*P* = 0.979) of patients with stage 1 cancer, which was also observed among patients with stage 2 or stage 3 cancer. Ultimately, ligation level was not an independent predictive factor for either OS or RFS.

**Conclusions:**

HT resulted in a significantly higher incidence of anastomotic fistula and worse preservation of urinary function. Level of IMA ligation did not significantly affect long-term outcomes of patients with sigmoid colon or rectal cancer after curative laparoscopic surgery.

## Background

Colorectal cancer (CRC) is the third most common cancer and causes the second most cancer-related deaths [1, 2]. Of all the colorectal cancers, rectal cancer and sigmoid colon cancer are the most common [3]. Curative surgery remains the basis of treating rectal cancer and sigmoid colon cancer. In 1908, Miles for the first time introduced the concept of en bloc removal of cancerous tissues and drainage lymph node systems [4]. Miles suggested that inferior mesenteric artery (IMA) should be dissected to the distal part of the branch of the left colic artery, which was referred to as a low tie (LT) procedure [4]. In the same year, another surgeon, Moynihan advocated that IMA should be dissected until its origin from the abdominal aorta had been exposed as well as dissection of apical lymph nodes [5], which was referred as to a high tie (HT) procedure [5].

HT has been advocated for curative resection and precise pathological staging [6–8]. Recently, LT has been recommended considering the fact that some studies report that LT is not significantly different from HT in terms of long-term survival [9–11]. Additionally, in some other studies, LT has been proposed due to the decreased blood flow to the proximal colon after HT [12–15]. Similarly, the American Society of Colon and Rectal Surgeons has proposed LT in the textbook of colon and rectal surgery considering the decreased blood supply in the proximal colon observed after HT, while HT should be performed among patients suspected to have involved lymph nodes around IMA or situations where extravascular dissection was needed to obtain additional proximal colon to avoid excessive tension of anastomosis [16]. According to Japanese guidelines on colorectal cancer, for patients with T2 or more advanced disease, lymph node dissection around IMA should be performed [17]. However, not so many studies definitely reporting the superiority of either ligation technique to the other one have been published. And several studies have reported that LT is not significantly different from HT in terms of short-term and long-term outcomes and in these studies; large-scaled randomized controlled trials are suggested [18–20]. Therefore, the clinical problem whether preserving blood supply to proximal colon by LT would decrease incidence of anastomotic fistula remains to be solved. Furthermore, effects of ligation level on other short-term results and long-term survival should also be evaluated. Thus, we performed the present study to evaluate whether level of IMA ligation would significantly affect short-term and long-term outcomes of patients with rectal or sigmoid colon cancer.

## Methods

### Patients

Medical records of patients with rectal or sigmoid colon cancer who had undergone curative laparoscopic surgery between January 2008 and December 2014 at the Department of Gastrointestinal Surgery, Guangdong Provincial Hospital of Traditional Chinese Medicine were retrospectively reviewed. The following clinicopathological variables were retrieved from their medical records: demographic variables, surgery-related variables, pathological variables, postoperative complications, adjuvant treatment, and follow-up variables.

Inclusion criteria were as follows: 20 years or older, pathologically confirmed adenocarcinoma of rectum or sigmoid colon, no distant metastasis, elective procedure, no history of abdominal surgery, and providing written informed consent. Clinical TNM staging was identified by combining results of colonoscopy, enhanced CT (thoracic, abdominal, and pelvic), and/or magnetic resonance imaging (MRI).

Exclusion criteria included the following: synchronous or metachronous malignant tumors of other organs, multiple colorectal cancer, acute intestinal obstruction or perforation, pregnant patients, and patients lost during early follow-up. General conditions of all the patients were evaluated preoperatively by an experienced anesthesiologist from our center. Abdominoperineal resection, rectal intersphincteric resection, and Hartmann^’^s operation were excluded from this study. This study was approved by the Ethical Committee of Guangdong Provincial Hospital of Traditional Chinese Medicine. All the patients had given his or her written informed consent. The Declaration of Helsinki was adhered to during the whole process of this study [21].

### Procedure

Surgeries were accomplished by advanced general surgeons specialized in colorectal cancer surgery. All these surgeons had at least 10 years of clinical experience in tertiary hospitals. All the surgeries were performed according to Chinese guidelines on colorectal cancer [22, 23].

Previously, for patients with a giant tumor (6 cm or larger), open surgery was preferred. However, in our study, all the patients underwent laparoscopic surgeries. All the laparoscopic surgeries were accomplished in a medial-to-lateral way. Firstly, we dissected lymph nodes around IMA and performed a retroperitoneal dissection. HT or LT was chosen by each patient after the surgeon in charge detailedly explained the advantages and disadvantages of HT and LT, unless there were clear contraindications or indications for LT. Situations where LT was specially contraindicated included the following two aspects: significant metastases to lymph nodes around the root of IMA and obvious invasion outside the serous layer. Patients who needed to undergo neoadjuvant chemoradiotherapy and those with poor nutritional status before surgery are suggested to undergo LT instead of HT since these patients were at significantly higher risk of anastomotic fistula. In HTG, the IMA was divided and ligated at its origin from the abdominal aorta, while for LTG, IMA was divided at the level of LCA branching, and a simultaneous lymph node dissection around IMA was performed. Secondly, the left colon was mobilized. Thirdly, we cut the distal rectum using a linear stapler after irrigating the rectum. Fourthly, the removed specimen was taken out via a small incision after the proximal colon was cut approximately 10 cm from the cancer. The proximal margin was at least 10 cm, while a distal margin of 3 cm was needed for cancer of the upper rectum and 2 cm for cancer of the lower rectum. The upper part of rectum was divided from the lower part of rectum by the peritoneal reflexion. Blood flow to the proximal colon stump in both arms was evaluated by a hemorrhage test of the marginal artery. The presence of palmic hemorrhage was indicative of sufficient blood supply. Resection of the proximal colon was not performed until confirmation of sufficient bleeding. Anvil of circular stapler was installed and fixed to the stump of the proximal colon. After the pneumoperitoneum was re-established, a pelvic sidewall lymphadenectomy was performed among patients with clinical T3 or deeper cancers with involvement of drainage lymph nodes identified by preoperative imaging examination. Finally, a double stapling technique was adopted to perform reconstruction. All the anastomotic reconstructions were accomplished in a straight fashion. After accomplishing reconstructions, we performed air leak tests to detect imperfections. Proximal colon of the anastomosis was closed using forceps after the circular stapler was fired. A small quantity of saline was put into the pelvic cavity, and appearance of a bubble around the anastomosis was tested by pumping in air from the anus. If a bubble was detected, the anastomosis would be reinforced by the suture.

Surgeons in charge would decide whether a diverting stoma should be constructed among patients with a narrow male pelvic, positive result of air leak test, and an anastomotic level lower than 5 cm from the anal verge. If a diverting stoma was not constructed, an intraluminal drainage tube would be inserted to decrease pressure within the anastomosis.

### Adjuvant therapy

All patients with stages 3 or 2 cancer with high-risk factors (such as microvascular invasion and nerve invasion) were recommended by surgeons in charge to undergo postoperative adjuvant chemotherapy. For stage 3 cancer, the regimen including oral fluoropyrimidine and oxaliplatin was preferred, while oral fluoropyrimidine was recommended for stage 2 cancer.

### Follow-up

Unless otherwise contraindicated, all the patients were instructed to attend a recommended follow-up. The follow-up plan was made according to the Chinese guideline on CRC [21, 22]. For stage 1 CRC, the follow-up took place every 6 months for 5 years. While for stages 2 and 3 CRC, the follow-up took place every 3 months for the first 3 years and then every 6 months for 2 more years. Five years after surgery, for stages 2 and 3 CRC, follow-up took place every 1 year for 5 years. The following aspects were included in follow-up regimens: physical examination, carcinoembryonic antigen (CEA) and cancer antigen 19-9 (CA-199), liver ultrasonography examination (stages 1 and 2), contrast-enhanced CT (every 1 year, for colon cancer) or MRI (for rectal cancer, every 1 year), and colonoscopy (every 1 year for 3 years). Recurrence was confirmed by combining medical history, physical examination, CT, or MRI examination, and histopathological examination was the ultimate diagnostic criterion whenever possible. Positron emission tomography-computed tomography (PET-CT) would be performed if recurrence was suspected but not confirmed by other examinations.

### Assessment parameters

Preoperative parameters included the following ones: sex, age, previous surgery history (mainly abdominal surgery), and concomitant diseases (such as hypertension and diabetes mellitus). Operation-related parameters included the following aspects: date of operation, operation time, estimated blood loss (EBL), level of IMA ligation, and blood transfusion. And parameters assessing cancer characteristics include the following: histological component, differentiation, depth of invasion, lymph node metastasis, and pTNM stage. Parameters assessing postoperative outcomes were as follows: early and late complications, degree of complications, ICU stay, and length of hospital stay after surgery. Complications occurring within 30 days after surgery were defined as early complications, while those occurring after 30 days were defined as late complications. Anastomotic fistula was detected by CT examination after detecting purulent discharge through the drainage tube or the presence of peritonitis. Complications were graded according to the Clavien–Dindo classification [24]. Overall survival (OS) was defined as the duration between curative surgery and death, while the time length between curative surgery and cancer recurrence was defined as relapse-free survival (RFS). Both OS and RFS were calculated by Kaplan-Meier analysis, and corresponding differences between LTG and HTG were compared using log-rank test. pTNM stages of all patients were reassessed according to the eighth edition of International Union Against Cancer/American Joint Committee on Cancer (UICC/AJCC) TNM staging system for colorectal cancer. And for patients included in this study, at least 12 lymph nodes should be obtained. The following two scoring systems were used to evaluate urinary performance: International Prostatic Symptoms Score (IPSS) and International Consultation on Incontinence Questionnaire (ICIQ). However, since most Chinese patients were conservative about sex and not willing to talk about their sexual performance, we could not accurately assess the impacts of ligation level on sexual performance.

### Statistical analysis

Statistical Product and Service Solutions 22.0 (SPSS22.0, SPSS Inc., Chicago, IL, USA) was used to perform statistical analyses. Continuous variables were demonstrated as mean ± standard deviation, while categorical variables were presented as frequencies and percentages. Differences in proportions were evaluated by chi-square test, while differences for continuous variables were assessed by independent-samples *t*-test. Additionally, binary logistic regression analysis was performed to identify independent predictive factors for anastomotic fistula. Cox regression analysis (both univariate and multivariate) was performed to identify independent predictive factors for OS and RFS. *P*-values less than 0.05 were recorded as statistically significant.

## Results

### Patients and baseline characteristics

**Fig. 1:**
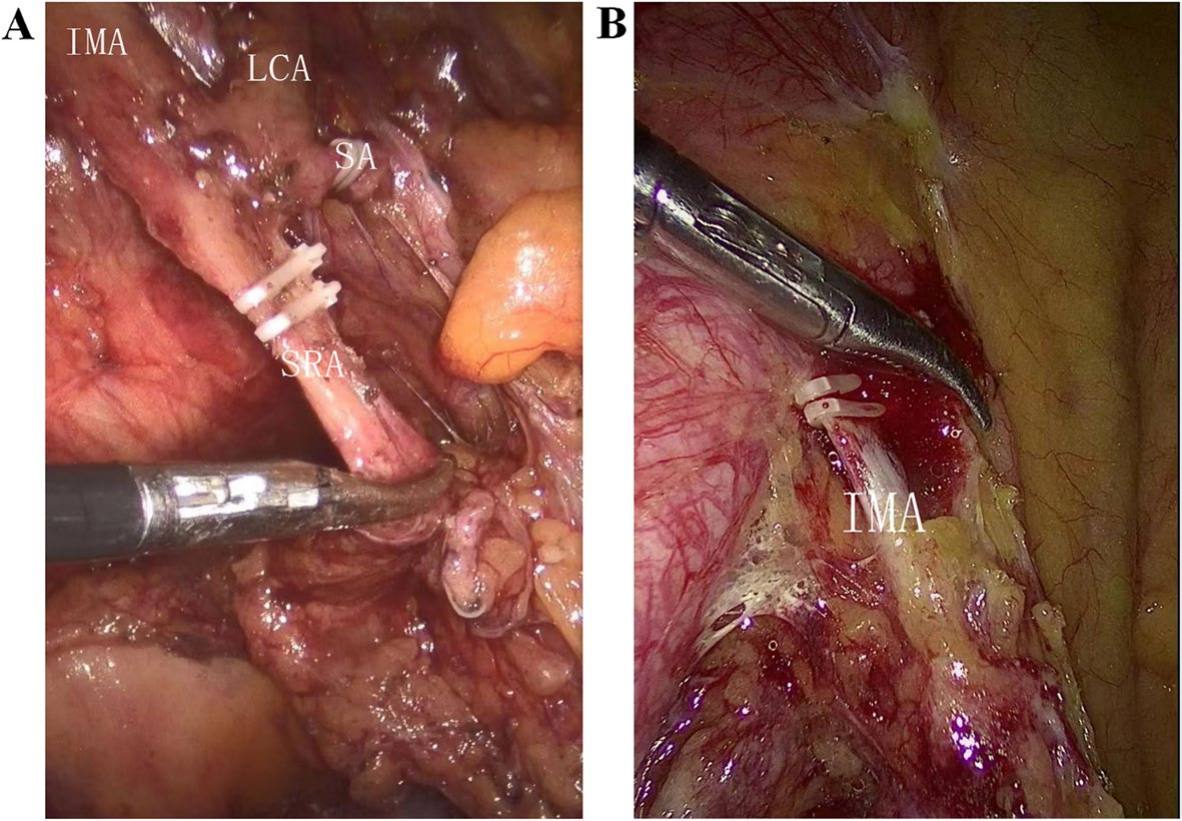
Representative images demonstrating HT and LT. **A** During LT, IMA, and LCA was skeletonized and preserved, while superior rectal artery (SRA) and sigmoid artery (SA) were ligated. Additionally, apical lymph nodes were dissected. **B** During HT, IMA was ligated at its origin with dissecting apical lymph nodes

A total of 283 patients were assigned into the HTG, while the LTG included 307 patients. Representative images showing LT and HT were demonstrated in Fig. 1. LTG was compared with the HTG regarding baseline characteristics, revealing that two groups were not significantly different in terms of these baseline characteristics (Table 1). For most patients in this study, one linear stapler cartridge was enough to transect the rectum.

**Table 1:** Comparisons between LT and HT regarding baseline characteristics

Characteristics	Ligation level		*χ*^2^/t	*p*-
	Low (*N* = 307)	High (*N* = 283)	Value	value
Age	58.47 ± 12.82	57.53 ± 12.36	0.908	0.364
≤ 65 y	209 (68.1%)	201 (71.0%)	0.603	0.437
> 65 y	98 (31.9%)	82 (29.0%)		
Gender			2.976	0.085
Male	175 (57.0%)	181 (64.0%)		
Female	132 (43.0%)	102 (36.0%)		
Diabetes and/or hypertension			2.435	0.119
Yes	47 (15.3%)	31 (11.0%)		
No	260 (86.7%)	252 (89.0%)		
Tumor size			0.595	0.441
≤ 5 cm	218 (71.0%)	209 (73.9%)		
> 5 cm	89 (29.0%)	74 (26.1%)		
Location			3.575	0.059
Sigmoid colon	109 (35.5%)	122 (43.1%)		
Rectum	198 (64.5%)	161 (56.9%)		
Gross morphology			0.632	0.729
Mass forming	97 (31.6%)	95 (33.6%)		
Ulcerative	174 (56.7%)	160 (56.5%)		
Infiltrative	36 (11.7%)	28 (9.9%)		
Differentiation			2.430	0.297
Well	18 (5.9%)	20 (7.1%)		
Moderate	257 (83.7%)	223 (78.8%)		
Poor	32 (10.4%)	40 (14.1%)		
Histological component			0.825	0.662
Adenocarcinoma	295 (96.1%)	268 (94.7%)		
Mucinous carcinoma	11 (3.6%)	13 (4.6%)		
Signet-ring cell carcinoma	1 (0.3%)	2 (0.7%)		
Vascular invasion			0.992	0.319
No	295 (96.1%)	267 (94.3%)		
Yes	12 (3.9%)	16 (5.7%)		
Nerve invasion			0.184	0.668
No	301 (98.0%)	276 (97.5%)		
Yes	6 (2.0%)	7 (2.5%)		
Depth of invasion			6.306	0.098
T1	12 (3.9%)	7 (2.5%)		
T2	72 (23.5%)	46 (16.2%)		
T3	141 (45.9%)	149 (52.7%)		
T4	82 (26.7%)	81 (28.6%)		
Lymph node metastasis			2.450	0.294
N0	189 (61.6%)	159 (56.2%)		
N1	71 (23.1%)	81 (28.6%)		
N2	47 (15.3%)	43 (15.2%)		
pTNM			5.285	0.071
I	69 (22.5%)	43 (15.2%)		
II	120 (39.1%)	116 (41.0%)		
III	118 (38.4%)	124 (43.8%)		
CEA level (μg/L)			0.653	0.419
≤ 5	205 (66.8%)	180 (63.6%)		
> 5	102 (33.2%)	103 (36.4%)		
Chemotherapy			2.059	0.151
No	170 (55.4%)	140 (49.5%)		
Yes	137 (44.6%)	143 (50.5%)		

### Short-terms outcomes

#### Anastomotic fistula

The incidence of anastomotic fistula was 7.42 (21/283) for HTG, while that for LTG was 3.58% (11/307). HTG had a significantly higher incidence of anastomotic fistula than LTG (*P* = 0.04, Table 2). Among patients in LTG experiencing anastomotic fistula, 7 ones had grade 2 anastomotic fistula, 3 ones grade with 3b anastomotic fistula, and 1 one with grade 5 anastomotic fistula (Table 3). Among patients in HTG experiencing anastomotic fistula, 15 ones had grade 2 anastomotic fistula, 5 ones grade with 4 anastomotic fistula, and 1 one with grade 5 anastomotic fistula (Table 3).

**Table 2:** Comparisons between LT and HT in terms of short-term outcomes

Characteristics	Low tie (*N* = 307)	High tie (*N* = 283)	*χ*^2^/t	*P*
Operation time	214.46 ± 83.90	216.90 ± 78.71	−0.365	0.715
Blood transfusion	3 (1.0%)	2 (0.7%)	0.000	1.000
Estimated blood loss	159.65 ± 29.65	157.11 ± 22.58	1.179	0.239
30-day mortality	1 (0.3%)	0 (0%)	0.000	1.000
ICU stay	3 (1.0%)	1 (0.4%)	0.177	0.674
Postoperative hospital stay (days)	11.06 ± 1.84	11.29 ± 1.72	−0.475	0.636
Bowel obstruction	5 (1.6%)	6 (2.1%)	0.194	0.659
Ileus	3 (1.0%)	5 (1.8%)	0.223	0.637
Anastomotic fistula	11 (3.6%)	21 (7.4%)	4.227	0.040
Surgical site infection	7 (2.3%)	13 (4.6%)	2.407	0.121
Number of retrieved lymph nodes	20.40 ± 8.32	20.89 ± 9.11	−0.673	0.501
Number of metastatic lymph nodes	1.55 ± 3.29	2.09 ± 5.08	−1.513	0.131

**Table 3:** Anastomotic fistula

Grade of leakage	Low tie (*N* = 307)	High tie (*N* = 283)
Grade 2	7 (2.3%)	15 (5.3%)
Grade 3a	0 (0%)	0 (0%)
Grade 3b	3 (1.0%)	0 (0%)
Grade 4	0 (0%)	5 (1.8%)
Grade 5	1 (0.3%)	1 (0.4%)
Total	11 (3.6%)	21 (7.4%)

### Other complications and operation-related parameters

Apart from comparing two groups regarding incidence of anastomotic fistula, we also assessed the impacts of ligation level on other complications, revealing that HTG was not significantly different from the LTG in terms of bowel obstruction (*P* = 0.659), surgical site infection (*P* = 0.121), and ileus (*P* = 0.637) (Table 2).

Additionally, two groups were compared regarding operation-related parameters, demonstrating that HTG was not significantly from LTG in terms of operation time (216.90 ± 78.71 vs 214.46 ± 83.90, *P* = 0.715), estimated blood loss (159.65 ± 29.65 vs 157.11 ± 22.58, *P* = 0.239), blood transfusion (*P* = 1.000), 30-day mortality (*P* = 1.000), ICU stay (*P* = 0.674), and hospital stays after operation (11.06 ± 1.84 vs 11.29 ± 1.72, *P* = 0.636) (Table 2).

### Independent predictive factors for anastomotic fistula identified by logistic regression analysis

In order to further evaluate the impacts of ligation level on anastomotic fistula, we then performed binary logistic regression analysis. Initially, it was demonstrated that gender (*P* = 0.034), location (*P* = 0.039), and ligation level (*P* = 0.040) were significantly associated with anastomotic fistula. Then, by accomplishing multivariate logistic regression analysis, we identified that location (*P* = 0.031, *OR* = 2.590, 95% *CI*: 1.092–6.143) and ligation level (*P* = 0.038, *OR* = 2.232, 95% *CI*: 1.047–4.758) were independent predictive factors for anastomotic fistula, which was demonstrated in Table 4

**Table 4:** Binary logistic regression performed to identify independent predictive factors for anastomotic fistula

Characteristics	Anastomotic fistula				Binary logistic regression analysis			
	Yes	No	*χ*^2^/t	*P*	*OR*	95% CI for Exp(B)		*P*
	(*N* = 32)	(*N* = 558)				Lower	Upper	
Gender			4.473	0.034	0.425	0.180	1.005	0.051
Male	25 (78.1%)	331 (59.3%)						
Female	7 (21.9%)	227 (40.7%)						
Age			0.009	0.925				
≤ 65	22 (78.1%)	388 (69.5%)						
> 65	10 (78.1%)	170 (30.5%)						
Diabetes and/or hypertension			0.154	0.695				
Yes	3 (9.4%)	75 (13.4%)						
No	29 (90.6%)	483 (86.6%)						
Tumor size			0.117	0.733				
≤ 5 cm	24 (75.0%)	403 (72.2%)						
> 5 cm	8 (25.0%)	155 (27.8%)						
Location			4.240	0.039	2.590	1.092	6.143	0.031
Sigmoid colon	7 (21.9%)	224 (40.1%)						
Rectum	25 (78.1%)	334 (59.9%)						
Ligation level			4.227	0.040	2.232	1.047	4.758	0.038
LT	11 (34.4%)	296 (53.0%)						
HT	21 (65.6%)	262 (47.0%)						

### Urinary performance assessed by IPSS and ICIQ

In order to further compare LTG and HTG in terms of short-term outcomes, we then reviewed the following two questionnaires: IPSS and ICIQ. HTG was not significantly different from LTG regarding preoperative IPSS (*P* = 0.107) and ICIQ (*P* = 0.269). Then, HTG was compared with LTG in terms of ICIQ (1 month after surgery) and IPSS (1 month after surgery), demonstrating that LT resulted in significantly better ICIQ (1 month after surgery) (*P* < 0.001), while LT was not significantly different from HT in terms of IPSS (1 month after surgery) (*P* = 0.961). However, 9 months after surgery, LTG was significantly better than HTG in terms of ICIQ (*P* < 0.001) and IPSS (*P* < 0.001). Therefore, it was revealed that LT was associated with significantly better preservation of urinary performance. Results of urinary performance assessment were presented in Table 5.

**Table 5:** Urinary performance assessed by IPSS and ICIQ

Characteristics	Low tie (*N* = 307)	High tie (*N* = 283)	*t*	*P*
ICIQ (preop)	2.71 ± 0.693	3.00 ± 0.667	−1.637	0.107
ICIQ (1 month)	3.61 ± 0.761	5.11 ± 0.685	−7.896	0.000
ICIQ (9 months)	3.06 ± 0.727	5.86 ± 0.651	−15.478	0.000
IPSS (preop)	14.19 ± 1.195	13.86 ± 1.113	1.115	0.269
IPSS (1 month)	18.16 ± 1.344	18.18 ± 1.335	−0.049	0.961
IPSS(9 months)	16.16 ± 1.573	20.79 ± 1.449	−11.704	0.000

### Long-term outcomes

#### Survival rate

**Fig. 2:**
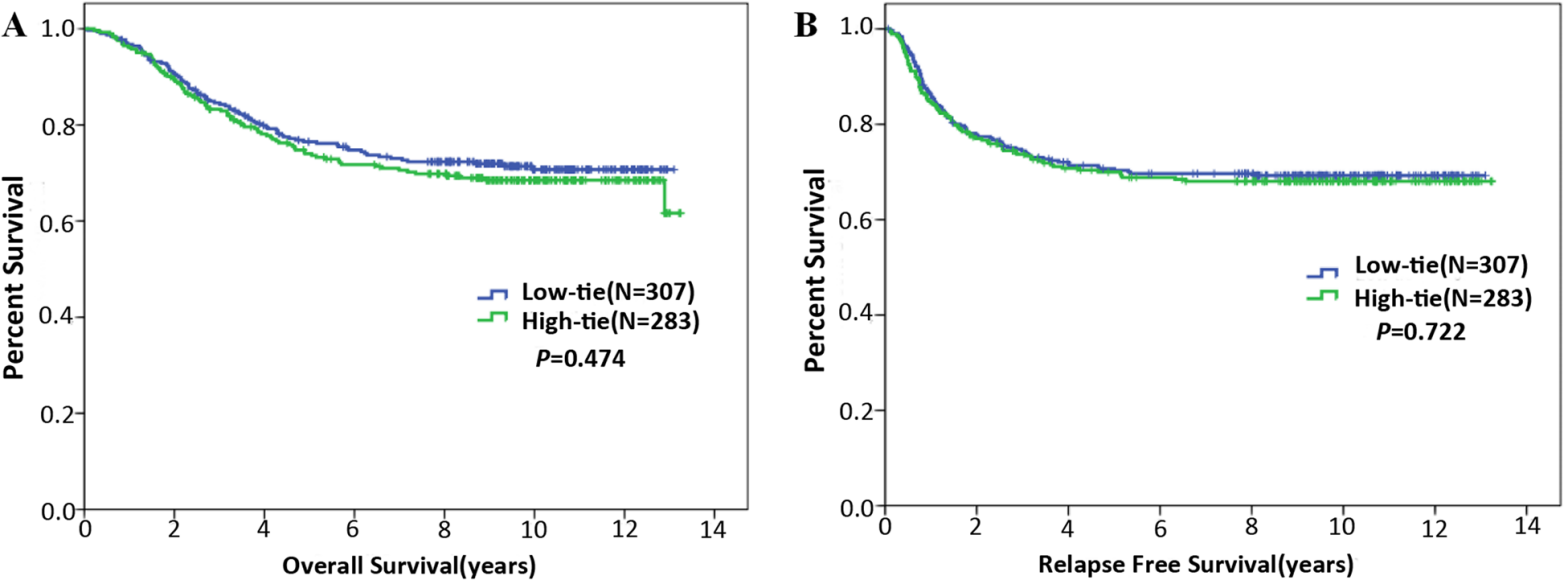
Kaplan-Meier analyses performed to assess the impacts of ligation level on OS and RFS. **A** Ligation level did not significantly affect OS. **B** Ligation level did not significantly affect RFS

**Fig. 3:**
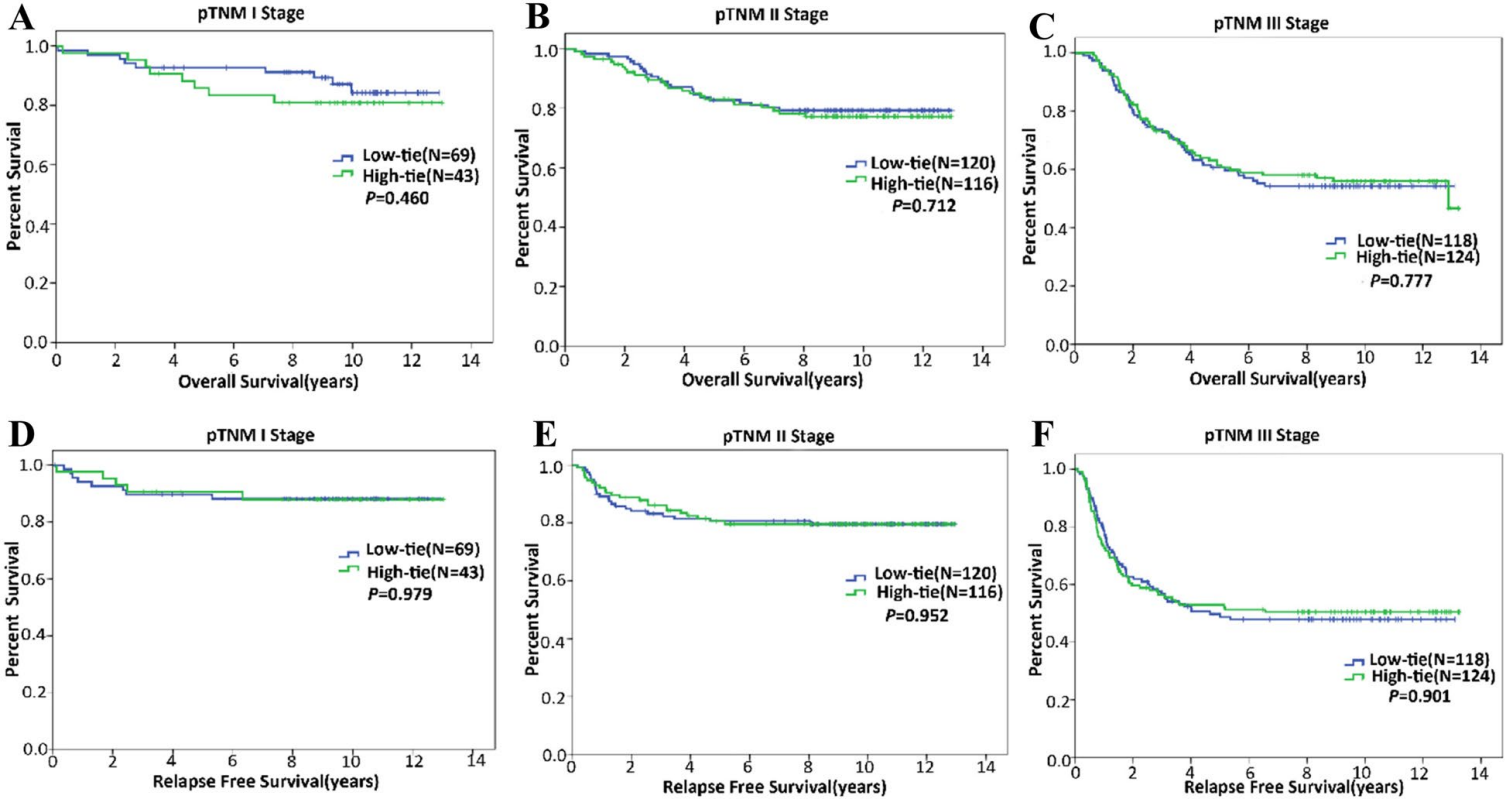
Kaplan-Meier analyses performed to assess the impacts of ligation level on OS and RFS of patients with cancer of different stages. **A** Ligation level did not significantly affect OS of patients with stage 1 cancer. **B** Ligation level did not significantly affect OS of patients with stage 2 cancer. **C** Ligation level did not significantly affect OS of patients with stage 3 cancer. **D** Ligation level did not significantly affect RFS of patients with stage 1 cancer. **E** Ligation level did not significantly affect RFS of patients with stage 2 cancer. **F** Ligation level did not significantly affect RFS of patients with stage 3 cancer

Five-year survival rates of HTG and LTG were 74.0% and 76.5% (*P* = 0.509), respectively. Five-year RFS rates of HTG and LTG were 70.0% and 70.3% (*P* = 0.917), respectively. Then, we performed Kaplan-Meier analysis to further evaluate the impacts of ligation level on long-term outcomes, revealing that HTG was not significantly different from LTG regarding OS (*P* = 0.474) and RFS (*P* = 0.722) (Fig. 2). Similarly, for patients with stage 1 cancers, ligation level did not significantly affect either OS (*P* = 0.460) or RFS (*P* = 0.979) (Fig. 3), which was also demonstrated among patients with stage 2 (Fig. 3) or stage 3 cancer (Fig. 3).

### Independent predictive factors for OS and RFS identified by Cox regression analysis

In order to further evaluate the impacts of ligation level on OS and RFS, we then accomplished Cox regression analysis. Initially, univariate Cox regression was performed to identify variables significantly associated with OS, demonstrating that age (*P* = 0.007, *HR* = 1.527, 95% *CI*: 1.125–2.074), differentiation (*P* < 0.001, *HR* = 0.291, 95% *CI*: 0.211–0.400), histological component (*P* < 0.001, *HR* = 2.291, 95% *CI*: 1.483–3.539), T stage (*P* < 0.001, *HR* = 1.581, 95% *CI*: 1.285–1.946), N stage (*P* < 0.001, *HR* = 2.059, 95% *CI*: 1.718–2.469), pTNM (*P* < 0.001, *HR* = 2.126, 95% *CI*: 1.681–2.688), vascular invasion (*P* < 0.001, *HR* = 4.157, 95% *CI*: 2.603–6.638), nerve invasion (*P* = 0.002, *HR* = 3.063, 95% *CI*: 1.506–6.233), and CEA (*P* = 0.008, *HR* = 1.505, 95% *CI*: 1.112–2.035) were significantly associated with OS (Table 6). Subsequently, those variables significantly associated with OS mentioned above were included in multivariate Cox regression analysis to determine independent predictive factors for OS, revealing that age (*P* < 0.001, *HR* = 2.011, 95% *CI*: 1.464–2.763), differentiation (*P* < 0.001, *HR* = 0.436, 95% *CI*: 0.288–0.662), N stage (*P* = 0.007, *HR* = 1.577, 95% *CI*: 1.133–2.196), TNM stage (*P* = 0.038, *HR* = 1.609, 95% *CI*: 1.344–2.079), and vascular invasion (*P* = 0.029, *HR* = 1.832, 95% *CI*: 1.063–3.159) were independent predictive factors for OS (Table 6).

**Table 6:** Cox proportional-hazard regression for OS

Characteristics	Univariate analysis				Multivariate analysis			
	*p*-Value	HR	95% CI for Exp(B)		*p*-Value	HR	95% CI for Exp(B)	
			Lower	Upper			Lower	Upper
Gender	0.659	1.071	0.791	1.449				
Age	0.007	1.527	1.125	2.074	0.000	2.011	1.464	2.763
Tumor size	0.265	0.819	0.577	1.163				
Gross morphology	0.813	1.029	0.811	1.307				
Differentiation	0.000	0.291	0.211	0.400	0.000	0.436	0.288	0.662
Histological component	0.000	2.291	1.483	3.539				
T	0.000	1.581	1.285	1.946				
N	0.000	2.059	1.718	2.469	0.007	1.577	1.133	2.196
pTNM	0.000	2.126	1.681	2.688	0.038	1.609	1.344	2.079
Vascular invasion	0.000	4.157	2.603	6.638	0.029	1.832	1.063	3.159
Nerve invasion	0.002	3.063	1.506	6.233				
CEA	0.008	1.505	1.112	2.035				
Ligation level	0.475	1.115	0.827	1.503				

In a similar way, we then accomplished Cox regression analysis to identify independent predictive factors for RFS. Initially, univariate Cox regression analysis was performed to identify variables significantly associated with RFS, demonstrating that differentiation (*P* < 0.001, *HR* = 0.317, 95% *CI*: 0.232–0.433), histological component (*P* = 0.008, *HR* = 1.849, 95% *CI*: 1.178–2.901), T stage (*P* < 0.001, *HR* = 1.680, 95% *CI*: 1.371–2.060), N stage (*P* < 0.001, *HR* = 2.343, 95% *CI*: 1.965–2.793), pTNM (*P* < 0.001, *HR* = 2.690, 95% *CI*: 2.102–3.443), vascular invasion (*P* < 0.001, *HR* = 4.328, 95% *CI*: 2.764–6.779), nerve invasion (*P* < 0.001, *HR* = 4.146, 95% *CI*: 2.251–7.636), and CEA (*P* = 0.034, *HR* = 1.378, 95% *CI*: 1.025–1.852) were significantly associated with RFS (Table 7). Subsequently, those variables significantly associated with RFS mentioned above were included in multivariate Cox regression analysis, revealing that differentiation (*P* = 0.002, *HR* = 0.548, 95% *CI*: 0.374–0.802), N stage (*P* = 0.003, *HR* = 1.598, 95% *CI*: 1.168–2.186), TNM stage (*P* = 0.028, *HR* = 1.897, 95% *CI*: 1.248–2.257), and nerve invasion (*P* = 0.047, *HR* = 1.946, 95% *CI*: 1.010–3.751) were independent predictive factors for RFS (Table 7).

**Table 7:** Cox proportional-hazard regression for RFS

Characteristics	Univariate analysis				Multivariate analysis			
	*p*-Value	HR	95% CI for Exp(B)		*p-Value*	HR	95% CI for Exp(B)	
			Lower	Upper			Lower	Upper
Gender	0.891	1.021	0.759	1.373				
Age	0.174	1.237	0.910	1.680				
Tumor size	0.109	0.753	0.532	1.066				
Gross morphology	0.810	1.029	0.815	1.299				
Differentiation	0.000	0.317	0.232	0.433	0.002	0.548	0.374	0.802
Histological component	0.008	1.849	1.178	2.901				
T	0.000	1.680	1.371	2.060				
N	0.000	2.343	1.965	2.793	0.003	1.598	1.168	2.186
pTNM	0.000	2.690	2.102	3.443	0.028	1.897	1.248	2.257
Vascular invasion	0.000	4.328	2.764	6.779				
Nerve invasion	0.000	4.146	2.251	7.636	0.047	1.946	1.010	3.751
CEA	0.034	1.378	1.025	1.852				
Ligation level	0.722	1.054	0.788	1.410				

## Discussion

The optimal level ligating IMA has been a controversial topic for more than 100 years and remains to be solved [8–12]. The controversies lie in whether long-term outcomes and short-term outcomes would be affected by ligation level. In studies recommending HT ligation, a curative therapy and accurate staging could be achieved, and both long-term outcomes and short-term outcomes are excellent. However, in studies supporting LT ligation, excellent blood flow to the proximal colon is the most important characteristics of LT ligation, and equivalent long-term survival is also achieved. Both HT and LT proponents have reached an opposing conclusion to the other side. Therefore, this study was performed to solve this controversy.

Firstly, we compared LT with HT in terms of anastomotic fistula, demonstrating that HT resulted in a higher incidence of anastomotic fistula and HT was an independent predictive factor for anastomotic fistula, which was consistent with some previous studies. Despite the fact that incidence of anastomotic fistula is not solely affected by the blood flow from the inferior mesenteric artery, blood flow to anastomosis, however, is considered as the most important factor. In theory, blood flow to anastomosis is significantly better preserved after LT than that after HT. As a matter of fact, it was reported by several previous studies that in comparison with LT, HT resulted in lower blood flow to the proximal colon [12, 14, 15]. However, several studies reported that HT did not increase the risk of anastomotic fistula [25–27]. In a few studies, several tests were reported to assess blood flow to proximal colon. In the study by Fujii S. et al., the authors performed a hemorrhage test to decide a proximal portion of colonic stump before anastomosis [28], which, however, was not a quantitative method, and they argued that quantitative methods should be developed to more accurately assess blood flow to the proximal colon. Some other studies reported that intraoperative fluorescence angiography using indocyanine green (ICG) was efficient in evaluating blood flow to the proximal colon [29–32]. More quantitative methods should be designed, helping surgeons assess perfusion of the proximal colon during surgery for rectal or sigmoid colon cancer.

However, decreased blood flow to anastomosis was not the only factor leading to anastomotic fistula. Other factors such as male gender and distance between anastomosis and anal verge were also risk factors for anastomotic fistula [28]. Thus, technical factors are involved in occurrence of anastomotic fistula but not all. Additionally, low perfusion to the distal rectum and high tension around the anastomosis were also factors contributing to anastomotic fistula. Therefore, we could conclude that multiple factors led to anastomotic fistula. It is not difficult for us to understand why in some studies ligation level does not significantly affect the incidence of anastomotic fistula. Subsequently, we evaluated the effects of ligation level on other complications and revealed that ligation level did not significantly affect occurrence of other complications except the better preservation of urinary performance after LT than after HT. According to a study by Fujii S. et al., HT led to a significantly higher incidence of bowel obstruction than LT [28]. Fujii S. et al. speculated that wider dissected range of retroperitoneal surface in HT was the possible reason [28]. In our opinion, the higher incidence of bowel obstruction after HT reported by Fujii S. et al. was the result of wider injury to pelvic nerves which was also reason why HT led to worse urinary performance. Consistent with some other studies, we advocated LT since it was indeed demonstrated that LT resulted in significantly less anastomotic fistula, and it was not so complex. Some marginal arteries between superior mesenteric artery (SMA) and IMA have been proposed. These marginal arteries included Riolan arch, Drummond artery, and Moskowitz artery [33–35], which have been thought as the basis of avoiding anastomotic fistula after HT. However, existence of these marginal arteries is still a controversy [33–35]. Therefore, we need to design methods assessing distribution of collateral artery between IMA and SMA since knowing the distribution of these collateral arteries could help us choose the reasonable ligation level. A few methods assessing distribution of collateral artery between IMA and SMA have been reported. Some studies reported that intraoperative fluorescence angiography using indocyanine green (ICG) was efficient in evaluating blood flow to the proximal colon [29–32]. CT angiography-based small vessel imaging has also been investigated in surgery for rectal or sigmoid colon cancer [33, 36]. Therefore, in our opinion, preoperative CT angiography or intraoperative fluorescence angiography using indocyanine green should be performed to evaluate the distribution of collateral artery between SMA and IMA to identify patients without adequate collateral artery between SMA and IMA who may have a higher incidence of anastomotic fistula. And these patients should undergo LT rather than HT, which, however, should be further investigated by more in-depth studies.

We also evaluated the impacts of ligation level on urinary performance, demonstrating that LT resulted in better preserved urinary performance. However, since most Chinese patients are quite conservative about their sexual performance, we could not accurately assess the impacts of ligation level on sexual performance. However, some previous studies had reported that LT resulted in significantly better preserved sexual and urinary performance. Mari GM et al. reported that LT led to better genitourinary function preservation without affecting initial oncological outcomes [37]. According to Si MB et al., LT was associated with a lower incidence of leakage and urethral dysfunction [38]. However, by far, studies comparing LT and HT regarding sexual and urinary performance are still scarce, and in these studies, the number of included patients is rather small, suggesting more randomized prospective studies including a larger number of patients are still needed. Actually in the study by Mari GM et al., both LT and HT led to impaired sexual and urinary performance, while sexual and urinary performance after LT improved more significantly than that after HT [37]. However, in two other studies, HT was not significantly different from LT in terms of male genitourinary function of patients with sigmoid colon cancer [39, 40]. Interestingly, LT was not significantly different from HT in terms of male genitourinary function, while LT was superior to HT in terms of sexual and urinary performance. Thus, rectal cancer seems to be different from sigmoid colon cancer regarding abnormal genitourinary function after curative surgery. However, in our study, HTG was not significantly different from LTG in terms of tumor location, suggesting that the finding that LT was associated with better preserved urinary function than HT was reliable. As known to us, injury to pelvic orthosympathetic nerve is the main cause of abnormal urinary and sexual function, suggesting that both LT and HT could cause injury to pelvic orthosympathetic nerve. And we speculate that in comparison with HT, LT causes less severe injury to pelvic orthosympathetic nerve. However, direct evidences supporting this speculation are still warranted. Additionally, studies solely including sigmoid colon cancer or rectal cancer that compared LT and HT in terms of sexual and urinary performance are still needed.

In addition to short-term outcomes, we also evaluated the effects of ligation level on long-term survival, demonstrating that neither OS nor RFS was significantly affected by ligation level. Additionally, it was also revealed that ligation level did not significantly affect long-term outcomes of patients with cancer of different stages, which was consistent with many other studies. Boström P. et al. reported that ligation level did not influence any patient-oriented oncological outcome [41]. Luo Y. et al. reported that LT was not significantly different from HT in terms of 3- and 5-year overall and disease-free survival [42]. According to AlSuhaimi M. A. et al., low IMA ligation with dissection of LNs around the IMA origin showed no differences in anastomotic leakage rate compared with high IMA ligation, without affecting oncologic outcomes [43]. Thus, LT plus lymphadenectomy around IMA could offer comparable OS and RFS to HT. Like in these studies, we also compared the number of dissected lymph nodes of two groups and revealed that HTG was not significantly different LTGs, which was consistent with a study by Olofsson F. et al. [44]. Thus, considering all these findings, we may conclude that HT was not significantly different from LT in terms of long-term outcomes and short-term outcomes except anastomotic fistula and urinary performance. However, patients with sigmoid colon cancer or rectal cancer had better undergo LT rather than HT, especially those with high risk of anastomotic fistula identified by preoperative CT angiography or intraoperative fluorescence angiography using indocyanine green.

However, this study also has some shortcomings. Firstly, this study was a retrospective one in nature, and prospective studies are needed to assess the impacts of ligation level on short-term outcomes and long-term outcomes. Secondly, this study included not so many patients as in other studies, warranting studies including more patients. Thirdly, this study included patients having undergone neoadjuvant chemotherapy or radiotherapy and those who had not, suggesting studies investigating the impacts of ligation level on outcomes of patients undergoing neoadjuvant chemotherapy or radiotherapy are warranted. Therefore, findings of this study could not be directly applied among patients who had undergone neoadjuvant chemotherapy or radiotherapy. As a matter of fact, patients having undergone neoadjuvant chemotherapy or radiotherapy were at risk of postoperative complications, and LT rather than HT should be performed to minimize the risk of complications especially anastomotic fistula. Fourthly, patients in this study had different backgrounds from those in studies performed in other countries. Studies including patients from Western countries should be performed. Fifthly, we did not perform CT angiography or intraoperative fluorescence angiography to assess the anatomical variance of left colonic artery as suggested by other studies. According to a study by Cirocchi R. et al., knowing the anatomical variance of left colonic artery is of paramount importance when performing a left colonic resection for either cancer or benign conditions such as diverticulitis [45]. Currently, a study based on preoperative CT angiography or intraoperative fluorescence angiography assessing the anatomical variance of left colonic artery is being carried out, and we will publish these results when this study is accomplished. However, despite these drawbacks, our study still could provide some useful suggestions for clinical practice since the optimal level ligating IMA is still a controversial topic in the area of general surgery.

In conclusion, for patients with sigmoid colon or rectal cancer, LT could efficiently prevent anastomotic fistula, and LT was associated with better urinary performance. LT was not significantly different from HT in terms of long-term outcomes. Despite the relative technical complexity of LT, we should perform LT as a routine operation for patients with rectal or sigmoid colon cancer especially for those with high risk of anastomotic fistula identified by preoperative CT angiography or intraoperative fluorescence angiography using indocyanine green. However, findings of this study should be assessed by more in-depth studies.

## Data Availability

The datasets generated and/or analyzed during the current study are not publicly available due to the fact that some patients in this study did not agree to make their information publicly available but are available from the corresponding author on reasonable request.

## Abbreviations

IMA: Inferior mesenteric artery
HTG: high tie group
LTG: Low tie group
SSI: Surgical site infection
OS: Overall survival
RFS: Relapse-free survival
CRC: Colorectal cancer
MRI: Magnetic resonance imaging
LCA: Left colic artery
CEA: Carcinoembryonic antigen
CA-199: Cancer antigen 19-9
PET-CT: Positron emission tomography-computed tomography
BMI: Body mass index
EBL: Estimated blood loss
UICC/AJCC: International Union Against Cancer/American Joint Committee on Cancer
SPSS 22.0: Statistical Product and Service Solutions 22.0
HR: Hazard ratio
CI: Confidential interval
ICG: Indocyanine green
IPSS: International Prostate Symptom Score
ICIQ: International Consultation on Incontinence Questionnaire
